# miR-124 represses the mesenchymal features and suppresses metastasis in Ewing sarcoma

**DOI:** 10.18632/oncotarget.14394

**Published:** 2016-12-31

**Authors:** Yunyun Li, Gaohai Shao, Minghua Zhang, Fengchen Zhu, Bo Zhao, Chao He, Zhongzu Zhang

**Affiliations:** ^1^ Department of Orthopedics, the Yongchuan Hospital of Chongqing Medical University, Chongqing 402160, PR China; ^2^ Department of Gynecology and Obstetrics, the Yongchuan Hospital of Chongqing Medical University, Chongqing 402160, PR China

**Keywords:** Ewing sarcoma, miR-124, SLUG, cyclin D2, mesenchymal features

## Abstract

Metastasis is the most powerful predictor of poor outcome of Ewing sarcoma (ES). Thus, identification of new molecules involved in tumor metastasis is of crucial importance to reduce morbidity and mortality of this devastating disease. In this study, we found that miR-124, a highly conserved miRNA, was suppressed in ES tissues and might be associated with tumor metastasis through suppressing its mesenchymal features. Overexpression of miR-124 suppressed the invasion of ES cells *in vitro* and tumor metastasis *in vivo*, which might be achieved through suppressing its mesenchymal features, as overexpression of miR-124 could repress the mesenchymal genes expression, and inhibit cell differentiation to mesenchymal lineages in ES cells. However, when SLUG was experimentally restored in these cells, mesenchymal features including suppressed expression of mesenchymal genes and decreased invasive ability were observed. We also found that cyclin D2 (CCND2) was a novel target gene of miR-124, and was directly involved in miR-124-mediated suppressive effects on cell growth. Lastly, we found that treatment with 5-Aza-CdR restored the expression of miR-124, accompanied with suppressed cell proliferation, invasion and mesenchymal features of ES cells, which demonstrated that hypermethylation might be involved in the regulation of miR-124 expression. Collectively, our data suggest that hypermethylation-mediated suppression of miR-124 might be involved in the tumor initiation and metastasis through suppressing the mesenchymal features of ES cells.

## INTRODUCTION

Ewing sarcoma (ES), characterized by the oncogenic fusion protein and transcription factor, EWS-FLI, is the second most common primary bone and soft malignant tumor of adenonts [1]. The presence of tumor metastasis is one of the most powerful predictors of poor outcome, which brings the overall survival rate from 70% to 75% for localized disease to less than 20% for those with metastasis [2]. Approximately 20% to 25% of the patients had detectable metastatic spreads at diagnosis [3]. Importantly, even patients lacking overt metastasis likely harbor micrometastases as indicated by the high rate of relapse following surgical resection of the primary tumor in the absence of systemic chemotherapy [4, 5]. Hence, identification of new signals involved in tumor metastasis is of crucial importance to reduce the morbidity and mortality of this devastating disease.

Metastasis is a multistep and complex process that results in the dissemination of cells from the site of primary tumor to distant organs [6]. For carcinomas, the procedure of epithelial to mesenchymal transition (EMT) plays a pivotal role in the tumor metastasis [7]; while, for sarcomas which were thought to arise from mesenchymal tissues, the effects of cellular plasticity transition on tumor metastasis remain less known. It is well established that ES cells have mesenchymal features, which become more pronounced with EWS-FLI knock-down [8]. Strikingly, overexpression of ZEB2 in ES cells, which is an EMT-inducing transcription factors (EMT-TF), could repress epithelial phenotypes and facilitate tumor metastasis [9], which suggests that the transition of cellular plasticity might participate in the tumor metastasis of ES.

Nowadays, microRNAs (miRNAs), which are a kind of small, endogenous, non-coding RNAs, are emerging as novel biomarkers of the disease and are considered to be critical components of cancer signaling network [10, 11]. By partially complementing with the 3′-untranslated region (3′UTR) of specific messenger RNAs (mRNAs), miRNAs can regulate translational efficiency or cleavage of target mRNAs and then modulate the target gene expression [12]. The loss of homeostasis in the miRNA/mRNA axis leads to relevant pathologic events, including the regulation of EMT [13, 14]. Among of which, miR-124 is an interesting one not only for its highly conserved and tissue-specific expression in nervous system [15], which has been reported to be another kind of cell origin of ES [16, 17], but also for its putative tumor-suppressive role in a variety of cancer, especially in the progression and metastasis of tumor [11]. In this paper, we want to identify the putative expression and significance of miR-124 in ES and relative mechanisms might be involved.

## RESULTS

### miR-124 expression is suppressed in ES tissues and related with tumor metastasis

**Figure 1:**
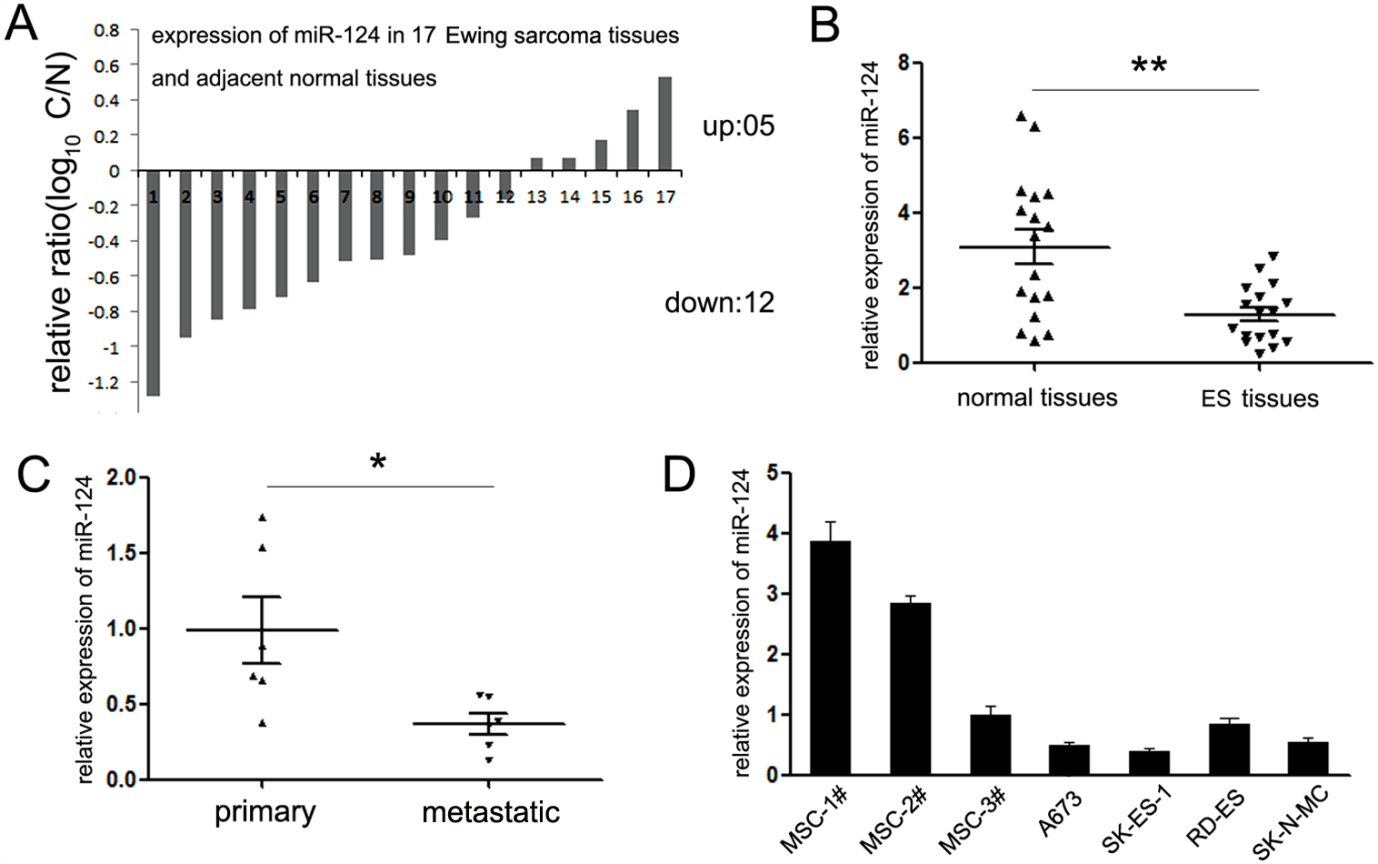
Expression of miR-124 in Ewing sarcoma specimens and cell lines **A**. The expression of miR-124 in 17 paired of ES tissues and adjacent normal tissues was detected by quantitative RT-PCR. 12 cases of ES tissues showed suppressed expression of miR-124. Data are shown as log_10_ of relative ratio change of ES tissues relative to adjacent normal tissues; **B**. Statistical analysis of relative mean level of miR-124 expression in ES tissues and adjacent normal tissues. The mean level of miR-124 is much lower in ES tissues; **C**. Statistical analysis of relative miR-124 expression levels in primary ES tissues compared to the metastatic lesions. The expression of miR-124 was suppressed in metastatic lesions of ES tissues; **D**. The expression of miR-124 in three ES cell lines (A673, SK-ES-1, SK-N-MC and RD-ES) was analyzed relative to human mesenchymal stem cells (MSCs). The expression of miR-124 was significantly suppressed in A673, SK-ES-1 and SK-N-MC relative to all MSCs; all *P<0.05. In RD-ES cells, miR-124 was significantly suppressed compared with MSC1# and MSC2#; *P<0.05, except for MSC3# (*P*>0.05). The data are representative of at least three independent experiments. Error bars represent s.e.m. **P<0.01.

In an attempt to explore the expression and significance of miR-124 in ES initiation and progression, we detected the expression of miR-124 in 17 paired of ES tissues and adjacent noncancerous tissues. Among these tissues, 12 cases of tumor tissues exhibited decreased expression of miR-124 (70%, 12 out of 17, Figure 1A). Moreover, comparing with the adjacent tissues, the mean level of miR-124 in ES tissues is much lower (Figure 1B). The similar generality was found in ES cell lines. As shown in Figure 1D, comparing with human mesenchymal stem cells (hMSCs), the putative origin cells of ES, miR-124 was significantly downregulated in majority ES cell lines (A673, SK-ES-1 and SK-N-MC), except for the RD-ES cells, which only showed slight suppression on miR-124 expression (Figure 1D).

Among the 17 ES patients, 5 had detectable metastatic spread at diagnosis. Thus, we further compared the expression of miR-124 between metastatic and non-metastatic patients. The miR-124 quantification in metastatic group showed no statistical significance comparing with the non-metastatic one (Supplementary Figure S1A). However strikingly, it showed significant lower level in metastatic lesions *versus* matched primary tumors (Figure 1C). From these analyses, we postulated that miR-124 might perform as a tumor suppressor in ES, and participate in the regulation of tumor metastasis.

### MiR-124 functions as a tumor suppressor in Ewing sarcoma

**Figure 2:**
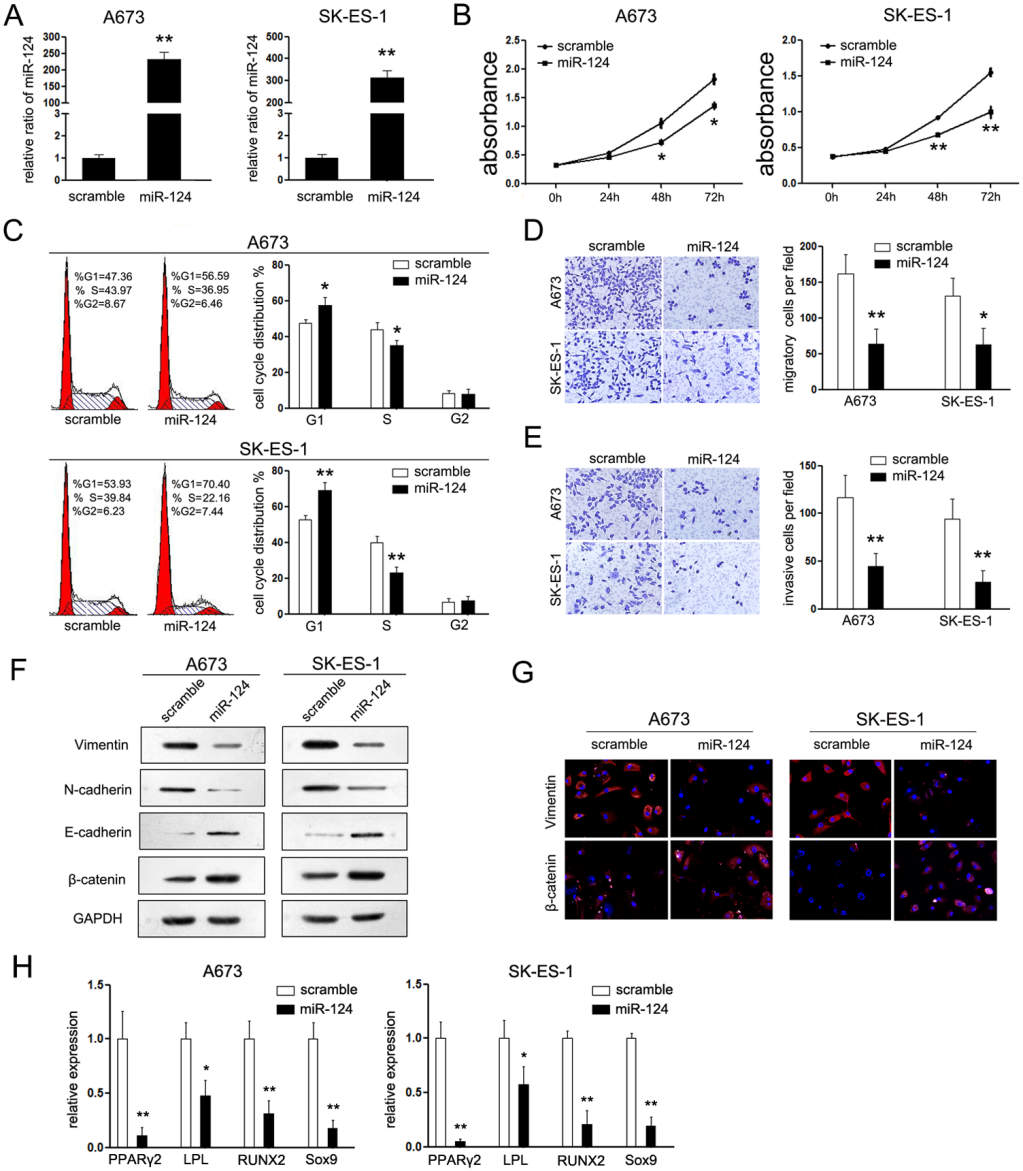
MiR-124 suppresses cell proliferation, migration, and mesenchymal features of ES cells *in vitro* **A**. RT-PCR was performed to detect the expression of miR-124 in ES cell lines (A673 and SK-ES-1) upon transfection with miR-124 mimic. Upon transfection with miR-124 mimic, the expression of miR-124 in both cell lines were increased; **B**. CCK-8 assays were performed to analyze the effect of miR-124 on cell proliferation of both cell lines. Transfection with miR-124 suppressed cell proliferation of both cells; **C**. Cell cycle analysis, by fluorescence-activated cell sorting (FACS) at 48h after transfection, were performed to analyze the effect of miR-124 on cell cycle progression of both cell lines. Upon transfection, the cell number of G1 phase was increased and which of S phase was decreased; **D, E**. The effects of miR-124 on cell migration and invasion were detected using trans-well chamber assays. Panel D showed the results on migration (×400); Panel E showed the results on invasion (×400). Cells transfected with miR-124 showed less cell number passing through the chambers in both conditions. **F**. Western blot analysis of the expression of epithelial and mesechymal marker proteins in ES cells transfected with miR-124 mimic was performed. **G**. Immunofluorescence staining assays were further used to detect the expression of Vimentin and β-catenin in ES cells. The expression of Vimentin (red) was suppressed in cells transfected with miR-124, while the expression of β-catenin (red) was increased in miR-124 group. **H**. RT-PCR was performed to detect the effects of miR-124 on the expression of PPAR2, LPL, RUNX2 and Sox9 upon transfection in different differentiation conditions. The data are representative of three independent experiments. Error bars represent s.e.m. *P<0.05; **P<0.01.

To test this hypothesis, we examined the phenotypic consequences of up-regulation and suppression of miR-124 in ES cells. Upon transfection with miR-124 mimic, the expression of miR-124 was up-regulated significantly (Figure 2A). Moreover, overexpression of miR-124 resulted in a significant decrease of cell proliferation (Figure 2B) associated with a simultaneous increased amount of G1-phase cells and a reduced number of cells in the S-phase of cell cycle (Figure 2C). Since the expression of miR-124 was suppressed in metastases of ES patients, we wanted to examine the effects of miR-124 on the metastatic potential of ES. Transwell matrigel migration and invasion assays were performed. Overexpression of miR-124 significantly inhibited cells passing through the trans-well chambers suggesting that transient miR-124 overexpression significantly inhibited the migratory and invasive capacity of A673 and SK-ES-1 cells *in vitro* (Figure 2D and 2E). On the other hand, inhibition of miR-124 by anti-miR-124 showed the opposite effects on the biological function of ES cells. As suppression of miR-124 resulted in increased cell growth and motility, upregulated number of cells in the S-phase of cell cycle (Supplementary Figure S2A-S2D), which further demonstrated the suppressive effects of miR-124 in ES.

### Inducible miR-124 expressing suppresses the mesenchymal features of ES cells

As mesenchymal features primes tumor cells to metastasize, we wondered whether it is involved in miR-124-mediated suppressive effects on ES metastasis. It is a pity that no difference was found between the morphology of ES cells with or without treatment of miR-124 (data not shown). However, the expression of related well-accepted epithelial and mesenchymal markers was varied upon transfection with miR-124 (30). Cells overexpressing miR-124 showed rearrangements from a mesenchymal to an epithelial-like state, which is confirmed by upregulated epithelial markers (E-cadherin and β-catenin) and downregulated mesenchymal markers (N-cadherin and Vimentin) (Figure 2F). Above results were further supported by immunofluorescence staining, as the expression of β-catenin was induced and localized to the plasma membrane in miR-124 treated cells. On the opposite, the mesenchymal marker Vimentin expression was notably decreased (Figure 2G).

It is suggested that miR-124 could inhibit the mesenchymal features of ES cells, thus we further investigated its effects on mesenchymal differentiation of ES cells. The A673 cells with stable miR-124 expression were established using lentiviral (Supplementary Figure S1B). PPARγ2 and LPL are two represent markers of the adipocyte lineage [18, 19]. Upon different differentiation conditions, A673 cells with stable expression of miR-124 exhibited a mild repression of PPARγ2 and a strong repression of LPL comparing with the control group (Figure 2H). In addition to the aforementioned suppression, restored expression of miR-124 also down-regulated the expression of RUNX2 and Sox9, which are specific markers for osteoblasts and chondrocytes, respectively [20, 21]. These results demonstrated that miR-124 inhibited cell differentiation to mesenchymal lineages, which strengthened the hypothesis that inducible miR-124 expressing suppresses the mesenchymal features in ES cells.

### SLUG is involved in miR-124-mediated suppression of tumor metastasis and mesenchymal features of ES cells

**Figure 3:**
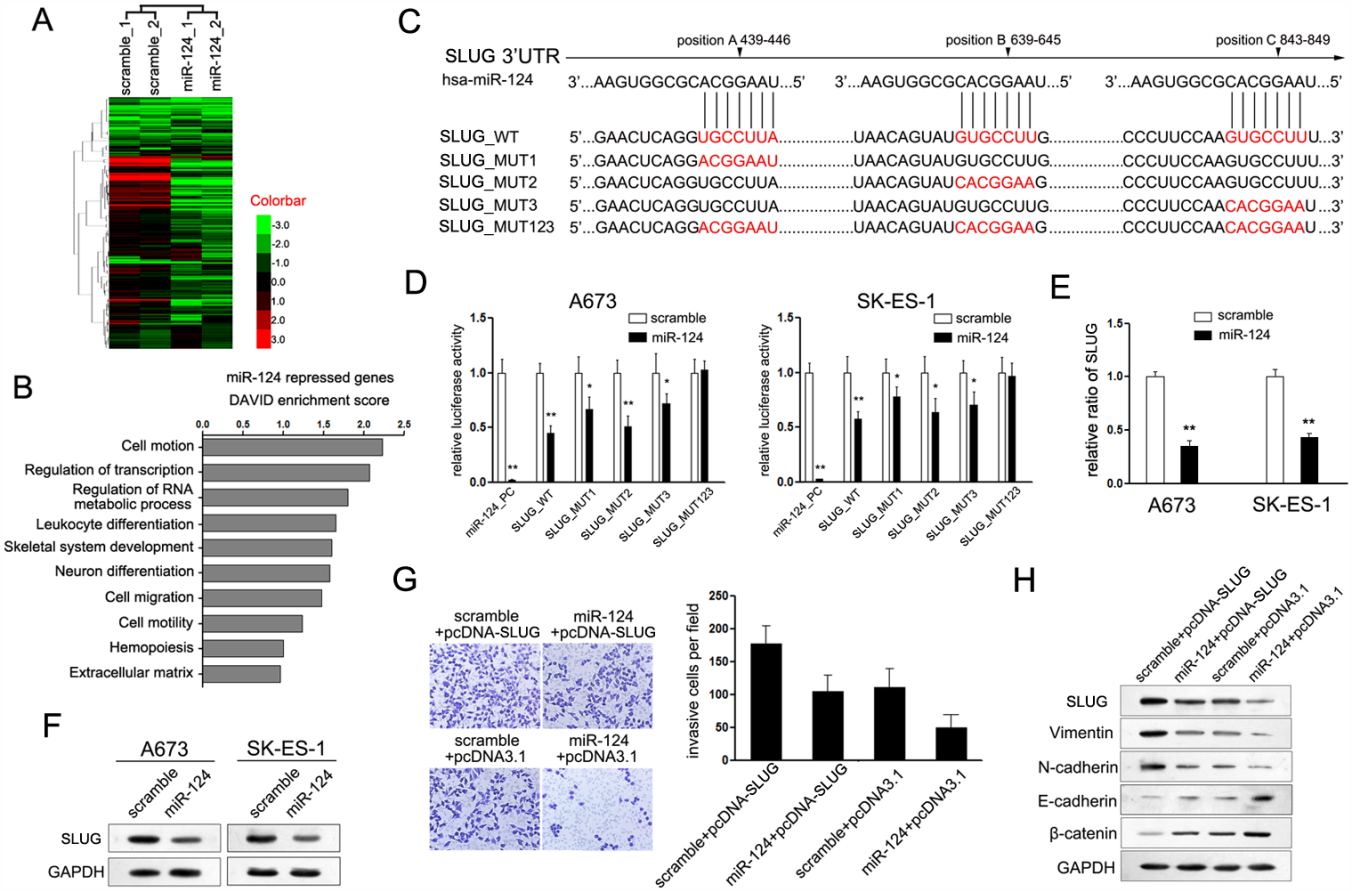
SLUG is involved in miR-124-mediated suppression of cell proliferation and invasion of ES cells **A**. Heat-map showing the expression profiles significantly differentially expressed in A673 and SK-ES-1 cells upon treatment with miR-124. **B**. DAVID analysis of the expression profiles present in A. **C**. Schematic representation of SLUG 3′UTR showing putative miR-124 target sites. There exist three binding sites of miR-124 in the 3′UTR of SLUG; **D**. Relative luciferase activity of the indicated SLUG reporter construct in ES cells, co-transfected with miR-124 mimic or scramble mimic, is shown. Transfection of miR-124 significantly repressed the luciferase activity of SLUG, while mutant of each site showed partly abrogates the repression; **E**. Quantitation RT-PCR of the expression level of SLUG mRNA in ES cells treated with miR-124 mimic. Overexpression of miR-124 suppressed the mRNA expression of SLUG; **F**. Western blot analysis showed the expression level of SLUG protein in ES cells treated with miR-124 mimic. Overexpression of miR-124 suppressed the protein level of SLUG; **G**. Transwell assays were performed to detect the effects of SLUG on cell invasion of ES cells that have been treated with miR-124 before (×400). Restored the expression of SLUG partially abolished the suppressive effects of miR-124 on the invasion of ES cells; **H**. Western blot analysis showed the expression levels of SLUG, E-cadherin, N-cadherin, Vimentin and β-catenin in ES cells treated as described in C.

Although miR-124 tunes expression of variety target genes, its mechanisms mediated in the regulation of ES cells remain less known. Thus, genome-wide gene expression profiling using RNA-sequencing (RNA-seq) in A673 and SK-ES-1 cells expressing miR-124 was performed (Figure 3A). Comparing with cells transfected with control mimic, 124 genes were found to be down-regulated by miR-124 overexpression. The significance cutoff was set at a 2-fold change and a false discovery rate (FDR) of 10%. To identify functional classes that were enriched in our miR-124 repressed gene list, we used the DAVID analysis functional annotation clustering algorithm [22, 23]. As shown in Figure 3B, the most enriched term representing this list was cell motion, followed by regulation of transcription and regulation of RNA metabolic process.

Among of which, SLUG (also named SNAI2), which was reported to be a suppressor of E-cadherin, attracted our attention most. E-cadherin is an important marker of epithelial phenotype, which means its suppression could induce the EMT progression. MiR-124 owns three predicted binding sites in the 3′UTR of SLUG mRNA as predicted (Figure 3C). All these targeting sites perform function, as miR-124 overexpression partly inhibited the transcriptional activity of each luciferase reporter containing mutant SLUG 3′UTR construct, respectively (Figure 3D). Overexpression of miR-124 reduced the expression of SLUG mRNA and protein in A673 and SK-ES-1 Ewing sarcoma cells (Figure 3E and 3F), while suppression of miR-124 up-regulated the expression of SLUG protein (Supplementary Figure S2E). These results demonstrated that SLUG was a target gene of miR-124 in ES cells.

Thus, we wondered whether SLUG is also involved in miR-124-mediated suppression on invasion and relative mesenchymal features of ES cells. Knocked down of SLUG through transfection with si-SLUG significantly inhibited the cell invasion of ES cells which phenocopied the phenotype induced by ectopic expression of miR-124 (Supplementary Figure S3C and S3D). Next, the rescue methodology was adopted to further examine the functional relevance of miR-124/SLUG interaction in A673 cells. We restored the expression of SLUG in cells transfected with miR-124 through transfecting the construct containing the ORF of SLUG gene. As expected, this construct rescued the level of SLUG in A673 cells that had been treated with miR-124 mimic for 24h before (Figure 3G). In agreement with the restored expression of SLUG, it partially abolished the suppressive effects of miR-124 on invasive capacity of A673 cells (Figure 3G). Moreover, the mesenchymal markers (Vimentin and N-cadherin) were positively regulated, whereas the epithelial markers (E-cadherin and β-catenin) were negatively regulated (Figure 3H). Altogether, the above results suggest that SLUG is a functional target of miR-124 contributing to its role in cellular plasticity-related migration and invasion in ES cells.

### miR-124 regulated cell cycle progression through inhibiting CCND2 and p-Rb

**Figure 4:**
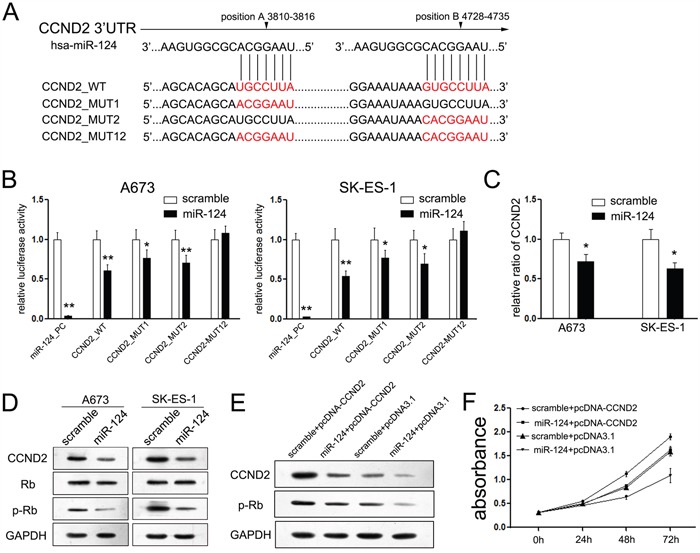
MiR-124 targets CCND2 gene in ES cells **A**. Schematic representation of CCND2 3′UTR showing putative miR-124 target sites. There exist two putative binding sites of miR-124 in the 3′UTR of CCND2; **B**. Relative luciferase activity of the indicated CCND2 reporter construct in ES cells, co-transfected with miR-124 mimics or scramble mimics, is shown. Transfection of miR-124 significantly repressed the luciferase activity of CCND2, while mutant of each site showed partly abrogates the repression; **C**. Quantitative RT-PCR assays were performed to detect the expression of CCND2 upon trasnfection with miR-124 mimic or scramble mimic. Transfection of miR-124 suppressed the mRNA level of CCND2 in ES cells; **D**. Western blot analysis showed the expression levels of CCND2, p-Rb and Rb proteins in ES cells treated with miR-124 mimic. The expression of CCND2 and its downstream gene p-Rb was suppressed upon transfection with miR-124, while the expression of Rb was not changed; **E**. Upon transfection with CCND2 construct, we rescued the expression of CCND2 in both ES cell lines; **F**. CCK-8 assays were used to detect to explore the effects of CCND2 on proliferation of ES cells that has been treated with miR-124 before; Restored the expression of CCND2 partially abolished the suppressive effects of miR-124 on proliferation of ES cells. The data are representative of three independent experiments. Error bars represent s.e.m. *P<0.05; **P<0.01.

The suppressive effects of miR-124 on the cell cycle transition from G1 to S phase promoted us to figure out the putative mechanism involved in miR-124-mediated regulation of cell cycle. Another down-regulated gene, CCND2 (cyclin D2) was picked out. CCND2 is one member of the cyclins that participate in promoting the cell cycle transition from G1 phase to S phase [24]. It revealed two putative miR-124-binding sites in the 3′UTR of the CCND2 promoter (Figure 4A), and both sites perform function in ES cells, as co-transfection with miR-124 and wild CCND2 construct significantly repressed the luciferase activity compared with the mutant groups (Figure 4B), while cells co-transfected with miR-124 mimic and each mutant construct (either mutant 1 or mutant 2 construct) exhibited partly inhibitory effect on luciferase activity compared with the pGL3-CCND2 3′UTR vector after miR-124 co-transfection, and the double mutant construct, Mut 12, showed complete reversal of inhibitory effect of miR-124 co-transfection (Figure 4B). Moreover, ectopic miR-124 expression significantly suppressed the mRNA and protein levels of CCND2 in both A673 and SK-ES-1 cells (Figure 4C, 4D), and inhibition of miR-124 up-regulated the protein level of CCND2 (Supplementary Figure S2E). It was reported that CCND2 regulated the cell cycle G1-to-S phase transition through regulating the phosphorylation level of Rb [25, 26]. Upon transfection with si-CCND2, the proliferation rates of ES cells were suppressed (Supplementary Figure S3A and S3B). To analyze whether miR-124 affects cell cycle progression through inhibiting CCND2/p-Rb axis, we measured the levels of p-Rb and total Rb upon transfection with miR-124 mimic. A decreased p-Rb level was detected in ES cells transfected with miR-124 mimic as compared to the cells transfected with the scramble mimic, while the level of total Rb remained consistent (Figure 4D).

We further restored the expression of CCND2 in A673 cells that have been treated with miR-124 before using another construct containing the full ORF of CCND2 gene (Figure 4E). In agreement with the restored expression of target proteins, increased cell growth (Figure 4F) was also observed in A673 cells transfected with CCND2 construct following the treatment of miR-124 mimic. These data indicated that miR-124 could inhibit cell proliferation through suppressing CCND2-mediated p-Rb activity.

### Ectopic expression of miR-124 impairs the formation of metastases of A673 cells *in vivo*

**Figure 5:**
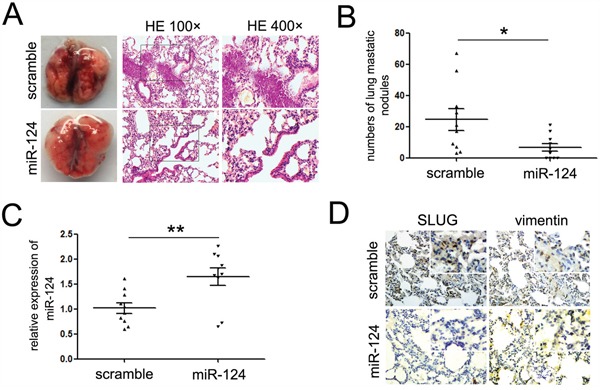
miR-124 suppresses the lung metastasis of ES cells *in vivo* **A**. Representative images of mouse lungs and histological inspection of mouse lungs for the presence of macrometastases and microscopic lesions after tail vein injection with A673 cells stably expressing miR-124 or scramble mimic lentiviral vector (×400). **B**. Quantification of lung metastasis nodules in lungs of both groups. Mice treated with miR-124 showed less numbers of lung metastatic nodules. **C**. Quantitative RT-PCR assays were performed to detect the expression of miR-124 in metastases derived from A673-miR-124 and A673 control groups. The expression of miR-124 was up-regulated in the metastases of mice treated with miR-124; **D**. Immunostaining assays were performed to explore the expression of SLUG and Vimentin in metastases treated as described in C. The expression of Vimentin was up-regulated, and the expression of was SLUG was decreased in the metastases of mice treated with miR-124. Representative images were shown. *P<0.05; **P<0.01.

We have established the ability of miR-124 to suppress migratory and invasive capacity, and repress the mesenchymal features in ES cells. We hypothesized that it might inhibit ES metastasis *in vivo*. To test this hypothesis, an orthotopic mouse metastasis model whereby cells are injected into tail veins and cells spontaneously metastasize was adopted. A673 cells with stable miR-124 expression (A673-miR-124 group) or empty lentiviral construct (A673-control group) were injected into the lateral tail veins of nude mice. 6-weeks post-injection, mice were sacrificed and the lungs were harvested. Mice in both groups showed evidence of visual macrometastases in the lung (Figure 5A). However, the number of lung metastasis nodules was significantly decreased in the A673-miR-124 group compared with the A673-control group (Figure 5B). Accordingly, we found that metastases derived from A673-miR-124 group had strong miR-124 expression (Figure 5C), while the expression of SLUG and Vimentin showed to be suppressed in metastases (Figure 5D). This strongly demonstrates that miR-124 suppresses tumor metastasis *in vivo*, and the suppression of mesenchymal features might be involved in the progression.

### MiR-124 expression is epigenetically regulated in ES

**Figure 6:**
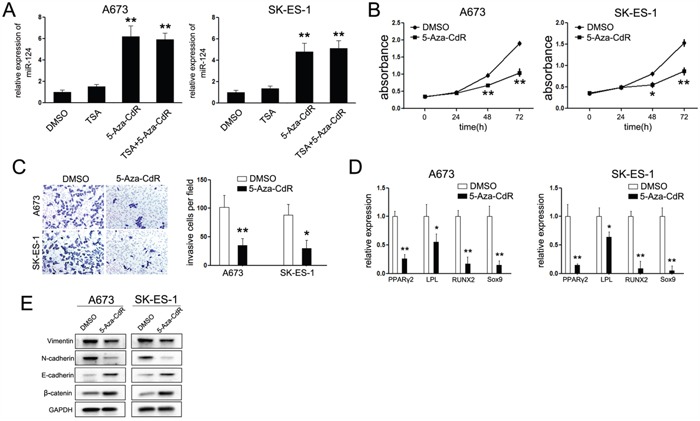
Treatment with 5-Aza-CdR duplicated the effects of miR-124 **A**. Relative expression of miR-124 upon treatment of 5-Aza-CdR and/or TSA. The expression of miR-124 was up-regulated in cells treated with 5-Aza-CdR but not TSA. **B**. CCK-8 assays analysis of proliferation rate of ES cells by 5umol/L 5-Aza-CdR. Treatment with 5-Aza-CdR suppressed the cell proliferation of ES cells; **C**. Trans-well chambers assays analysis of invasive ability of ES cells treated with 5umol/L 5-Aza-CdR. Cells treated with 5-Aza-CdR showed less number passing through the chambers; **D**. Western Blot analysis of specific epithelial and mesenchymal markers upon treatment with 5-Aza-CdR; **E**. Quantitative RT-PCR analysis of specific adipocytes, osteocytes and chondrocyte markers. The data are representative of three independent experiments. *P<0.05; **P<0.01.

Based on the above findings, we conclude that miR-124 was an important tumor suppressor in ES. However, the regulatory mechanisms of miR-124 expression in ES were still unknown. The CpG-rich regions for each genomic locus of miR-124 ((miR-124-1 (8p23.1), miR-124-2 (8q12.3), miR-124-3 (20q 13.33)) have been reported to be hypermethylated in many cancers [27–30]. To investigate whether miR-124 was epigenetically regulated in ES, A673 and SK-ES-1 cells were treated with dimethyltransferase inhibitor, 5-aza-2’-deoxycytidine (5-Aza-CdR) and the histone-deacetylase inhibitor trichostain A (TSA). As shown in Figure 6A, treatment of 5-Aza-CdR led to a dramatic upregulation of miR-124 compared with control groups. Moreover, treatment with 5-Aza-CdR resulted in a considerable decrease of cell proliferation associated with a reduced number of cells passing through the chambers in invasive assays (Figure 6B and 6C). Companied with the suppressed invasive capacity, the procedure of EMT was repressed, as the expression of epithelial markers was increased, while the expression of mesenchymal markers was down-regulated (Figure 6D).

Lastly, we further explored the effects of 5-Aza-CdR on cell differentiation. In the presence of 5-Aza-CdR, adipogenic differentiation medium led to a significant suppression of the adipogenic markers, PPARγ2 and LPL (Figure 6E). In the differentiate conditions for osteogenic and chondrogenic lineages, the aforementioned osteogenic marker, RUNX2, and chondrogenic marker, Sox9, were consistently suppressed upon treatment with 5-Aza-CdR (Figure 6E). These data suggest that epigenetic regulation is a pivotal mechanism involved in the regulation of miR-124 expression in ES, which might be involved in the tumor initiation and EMT-mediated suppression on tumor metastasis.

## DISCUSSION

Although miR-124 has been reported to be a tumor suppressor in many cancers, its definite function in ES has not been described yet. In this study, we found that miR-124 was suppressed in ES tissues and might be associated with tumor metastasis through suppressing the mesenchymal features.

MiR-124 is a highly conserved miRNA that tunes the expression of many genes. It used to be reported to perform an important role in neural progression, from nervous system development to normal neuronal cell function [15]. For example, overexpression of miR-124 could shift the gene expression profile of cervical HeLa cells towards a brain-like pattern [31], while in mice, knockdown of miR-124 results in defects of adult neurogenesis [32]. Recently, its suppressive effects on variety cancers were widely reported. Such as, our previous study found that it is suppressed in the endometrial endometroid adenocarcinoma, and its overexpression suppresses the malignant phenotype of EC cells [33]. Partially consistent with previous experiments, the expression of miR-124 was suppressed in ES tissues. Consistently, overexpression of miR-124 significantly suppressed cell proliferation, migration and invasion of ES cells *in vitro*, and tumor metastasis *in vivo*, while inhibition of miR-124 showed the opposite effects. These data demonstrated the tumor suppressor role of miR-124 in ES.

We further explored the relation between miR-124 with tumor metastasis. Although no statistical significance was found between the expression miR-124 in the metastatic and non-metastatic groups, which might be due to insufficient number of cases and lack of statistical power, significant lower level of miR-124 was found in the metastatic lesions of ES patients. Combined the factor that ES cells might be originated from neural crest or neuroepithelia-derived progenitor cell, which is the initial source of MSCs from mouse embryonic stem cells *in vitro* and showed highly conserved expression of miR-124 [34], with our results that miR-124 was suppressed in ES tissues, especially the metastatic lesions, we hypothesized that down-regulation of miR-124 might be involved in the initiation and progression of ES, and its correlating level might be changed in terms of tumor behavior and microenvironment, which means it might be regulated depending on epigenetic mechanisms. As expected, we found that the expression of miR-124 was restored upon treatment with 5-Aza-CdR. Strikingly, treatment with 5-Aza-CdR duplicated the suppressive effects of miR-124 on ES cells, which demonstrated that hypermethylation mediates the suppression of miR-124 in ES.

Metastasis is a complex process, which requires a tumor cell possess both epithelial and mesenchymal characteristics. Epithelial features promote cell growth at both the primary and secondary sites, while mesenchymal features contribute a migratory capacity to these cells facilitating escape from the primary site, the ability to survive in the circulatory, and extravasate at distant sites [35]. Recently, it was proposed that mesenchymal features prime the ES cell successfully metastasize, as they found that EWS-FLI translocation could block the mesenchymal differentiation of a cell that is undergoing normal developmental EMT procedure, and resulted in an undifferentiated ES cell [9]. Herein, we found that overexpression of miR-124 as well as treatment with 5-Aza-CdR suppressed the mesenchymal features of ES cells. Inducible miR-124 expressing suppressed the expression of mesenchymal markers, increased the expression of epithelial markers, suppressed tumor metastasis *in vitro* and *in vivo*, and inhibited cell differentiation to mesenchymal lineages. These data suggested that suppression of miR-124 might prime ES cells to metastasis through regulating the mesenchymal features of ES cells.

To further identify the putative mechanisms mediated the suppressive effects, we detected the genome-wide gene expression profiling upon transfection with miR-124. Among of which, 124 genes were down-regulated upon treatment with miR-124, and the most enriched functional class was cell motion. Among the repressed genes, SLUG attracted our attention most. SLUG is a member of the SNAIL family of zinc finger transcriptional repressor mediating sequence-specific interactions with DNA. The SNAIL family has been reported perform critical role in a variety of developmental and cellular processes, many of which relate to cell motility and induction of the EMT; these include but are not limited to: neural crest migration, wound re-epithelialization and tissue fibrosis in the adult [36–38]. Interacts with the CtBP1 co-repressor, SLUG suppresses the expression of epithelial marker, E-cadherin, and negatively regulates induction of the EMT [39]. In breast cancer, SLUG has been reported to be target gene of miR-124 and be involved in miR-124-mediated suppression of migration and EMT [40]. Our results also identified the target role of SLUG and the effects of miR-124/SLUG axis in ES cells. Restored the expression SLUG could partially abolish the suppressive effects of miR-124 on the migration and mesenchymal features of ES cells. Interestingly, expect for its pivotal role in regulation of EMT, SLUG is also reported to be required for neural crest cell differentiation [41]. The suppression of SLUG is consistent with aforementioned inhibition of cell differentiation to mesenchymal lineages in ES cells.

Although the DAVID analysis did not find the suppressive effects on cell cycle regulation, among the suppressed genes, we found that cell cycle related gene, CCND2 is a putative target gene of miR-124. CCND2 gene encoded a protein termed G1/S-specific cyclin-D2, which belongs to the highly conserved cyclin family. CCND2 forms a complex with CDK4 or CDK6 and functions as a regulatory subunit of them, whose activity regulates the phosphorylation of Rb and is required for cell cycle G1/S transition [42]. Herein, our data firstly elucidated that CCND2 is a novel target of miR-124 and loss of miR-124/CCND2 homeostasis contributed to ES progression. MiR-124 can regulate ES cell proliferation and G1/S transition through targeting CCND2, then modulating the level of p-Rb.

Recently, more and more studies have recognized that during some diseases, a full EMT is not always achieved. The phenotypic states of cells express the critical value for tumor growth and metastasis. A partial EMT, which means cells not only retain cell-cell junctions but also acquire invasive and motile properties, has significant potential to metastasize [43, 44]. For ES, the cells were stucked in between an epithelial differentiation state and a mesenchymal differentiation state, but with access to features of both cell types. This poorly differentiated cancer cells is poised for successful growth in the primary and secondary sites, owing to its epithelial features, while at the same time made competent for metastasis by its mesenchymal features [9, 45]. This may contribute to the limited latency seen in ES, as 72% of relapses occur within 2 years of diagnosis and 94% within 5 years [45]. Thus, the transition of mesenchymal features may offer a therapeutic opportunity for ES. In this study, miR-124 could suppress the migration and mesenchymel features of ES through targeting SLUG, and arrested the cell cycle progression through suppressing of CCND2. Expect for that, RUNX2 and Sox9 have also been reported to be the target genes [46, 47], which could explain the suppressive effects on cell differentiation, while another suppressed gene, PPAR2 and LPL were not the target genes of miR-124. As we have reviewed before, miR-124 was involved in several positive and negative regulatory circuits in the physiological and pathological processes [11], which means the putative suppressive effects on PPARγ2 and LPL might mediate through indirect mechanisms. More experiments are necessary to fully comprehend this in the future.

Taken together, our work demonstrated that hypermethylation-mediated miR-124 suppression participates in the initiation and metastasis of ES through regulating the translation of mesenchymal features, which may provide new ideas for ES therapy especially the metastatic ones. The deficiency of this experiment is that the *in vivo* work was only performed with A673 cells. It depends to say whether it performs function for other cell lines.

## MATERIALS AND METHODS

### Patients and tissue specimens

17 paired samples of human ES and their matched adjacent noncancerous tissues were collected at the time of surgery between 2002 and 2014 at Chongqing Medical University. Among the 17 ES patients, 5 patients had detectable metastatic spread at diagnosis, as 3 patients had bone marrow metastases, and 2 patients had lung metastases. The matched normal tissues were obtained 5 cm distant from the tumor margin, which were further confirmed by at least two pathologists. Upon resection, human surgical specimens were immediately frozen in liquid nitrogen and stored at -80°C in the refrigerator. All patients did not undergo any therapy before recruitment to this research. Use of the tissue samples for all experiments was approved by Ethics Committee of the instruction.

### Cell culture, transfection, treatment, differentiation and biological function assays

The relative materials and methods of cell culture, cell transfection, differentiation assays and relative biological function assays were described in the Supplementary File S1.

### RNA extraction and quantitative real-time PCR

For analysis the expression of miR-124 in ES, total RNA was isolated from cells and human surgical specimens according to the protocol of Recover All Total Nucleic Acid Isolation Kit (Ambion, Austin, TX, USA). Following gel electrophoresis verification of RNA integrity, total RNA was reverse transcribed using a First-Strand cDNA Synthesis kit (Invitrogen, Carlsbad, CA, USA) with specific primers as supplemented in Supplementary Table S1. The expression of small nuclear U6 was used as internal control. Then, qPCR was performed to quantify relative expression of miR-124 using the Quanti-Tect SYBR Green PCR mixture on an ABI PRISM 7900 Sequence Detection System (Applied Biosystems, Carlsbad, CA, USA). For analysis of miRNA, small nuclear U6 was used as internal control, while for analysis of mRNAs, GAPDH was used as the internal control. The primers of reverse transcription and qPCR were summarized in Supplementary Table S1. PCR efficiencies were calculated with a relative standard curve derived from a complementary DNA mixture and gave regression coefficients >0.95. The relative expression levels were evaluated using the 2^−ΔΔ*C*t^ method. All experiments repeated five times.

### RNA sequencing

RNA from A673 and SK-ES-1 cells transfected with miR-124 or control mimic 72 hours post-transfection was extracted using the RNAeasy kit (Qiagen) and treated with DNAse according to the manufacturer's instructions. To biological replicates, per condition were used to construct libraries for high-throughput sequencing and sequenced on the Illumina Hi-seq with 50 cycles of single end reads (Illumina, San Diego, CA, American). Sequence was aligned to the human genome build hg 19. Significance parameters were set at an FDR of 10% and 2-fold change.

### Luciferase reporter assays

To further identify the target role of SLUG and CCND2, the complete 3′UTRs of SLUG and CCND2 were amplified from genomic DNA and cloned into the pGL-3-vector (Promega, San Luis, CA, USA), respectively. The constructed 3′UTRs of both target genes were then mutated using the QuickChange Site-Directed Mutagenesis kit (Stratagene, Santa Clara, CA, USA). Cells were transfected with the pGL-3 firefly luciferase reporter (50 ng), pRL-TK Renilla luciferase reporter (10 ng), and the miR-124 or control mimic (50 nM). The luciferase reporter construct containing the miR-124 consensus target sequence served as positive control (PC), and the pRL-TK vector served as internal control. All transfections were carried out in triplicate using Lipofectamine 2000 (Invitrogen, USA). Cell lysates were prepared using Passive Lysis Buffer (Promega, San Luis, CA, USA) 48 h after transfection, and luciferase activity was measured using the Dual-Luciferase Reporter Assay (Promega, San Luis, CA, USA) and then normalized to Renilla luciferase activity.

### Plasmid construction

The full-length SLUG and CCND2 open reading frames were amplified and cloned into the pcDNA3.1 vector to generate pcDNA3.1-SLUG and pcDNA3.1-CCND2 constructs, respectively. Sequences of primers used for PCR amplification are summarized in Supplementary Table S1. The generated constructs of SLUG and CCND2 were then verified by sequencing (Beijing Tianyi Huiyuan Bioscience & Technology Inc., Beijing, China). The empty pcDNA3.1 construct was used as negative control. For rescue assays, ES cells were firstly transfected with miR-124 or scrambled mimic (60 nM) in six-well plates. 24 hours after transfection, cells were then co-transfected with pcDNA3.1-SLUG/CCND2 or pcDNA3.1 constructs (2.0 μg) and miR-124 mimic (30 nM). Cells were harvested at intervals and assayed.

### Westernblot analysis

For the westernblot assay, cells were harvested in ice-cold PBS 48h after transfection and lysed on ice in cold-modified radioimmunoprecipitation buffer supplemented with protease inhibitors. Protein concentration was determined using the BCA Protein Assay Kit (Bio-Rad, CA, USA) and equal amounts of protein were analyzed by SDS-PAGE. Gels were electroblotted onto nitrocellulose membranes (Millipore, WI, USA). After blocked with 5% non-fat dry milk in Tris-buffered saline containing 0.1% Tween-20 2 h, membranes were incubated at 4°C with primary antibodies (including anti-SLUG, anti-Vimentin, anti-E-cadherin, anti-N-cadherin, anti-β-catenin, anti-CCND2, anti-Rb, anti-p-Rb (Cell Signaling, USA) and GAPDH (Zhong-Shan JinQiao, China)) overnight. Then, membranes were incubated with respective second antibodies and detected by peroxidase-conjugated secondary antibodies using the enhanced chemiluminescence system (ECL) (Millipore, WI, USA). The experiment was repeated three times.

### lentiviral vector construction

The self-inactivating transfer vector pMIRNA1, control plasmid, and the packaging kit System Biosciences were used according to the manufacturer's instructions. DNA fragments 500 bp in size containing the miR-124 were inserted under the CMV promoter in pMIRNA1. After virus packaging, the recombinant lentivirus particles were harvested and titrated. The ES cells were infected with lentivector supernatants (lenti-miR-124 and lenti-control) at an MOI of 50 in the presence of polybrene (5 μg/mL). The cells were washed the next day with PBS and liquid cultures.

### Immunofluorescent staining

ES cells upon transfection were fixed with 4% paraformaldehyde at room temperature for 20 min, and washed in PBS for three times. Then, the cells were permeabilized with PBS containing 0.1% Triton X-100 (PBS-T) at room temperature for 90 min, and blocked with 1% BSA in PBS-T. Lastly, the cells were incubated with human anti-Vimentin and anti-β-catenin antibodies (1:200; Cell Signaling Technology Company, CSA) at 4°C overnight. The primary antibody was detected using anti-rabbit-Alexa 594-conjugated antibodies (1:200; Invitrogen). To detect nuclei, cells were co-stained with 4’, 6-diamidino-2- phenylindole. Fluorescence was observed and imaged using a Nikon Eclipse TE300 confocal laser microscope.

### *In vivo* metastasis assays

For the *in vivo* tumor metastasis, five-week-old nude male mice were used. All mice were maintained in the pathogen-free (SPF) conditions at our institution upon receiving. 2×10^6^ A673 cells with stable miR-124 expression were injected into the lateral tail veins of nude mice (12 per group). After 30 days, all mice were killed and the lungs were removed and fixed in 10% neutral phosphate-buffered formalin. The fixed samples were embedded in paraffin and stained with hematoxylin and eosin (HE). All animal experiments were housed and performed according to institute guidelines.

### Statistical analysis

Data were expressed as the mean ± standard deviation of at least three independent experiments. The Student's t-test was used for comparisons between two groups, and analysis of variance was used for comparisons among three groups. The chi-squared test was used for occurrence analysis. Statistical analysis was carried out using SPSS 15.0 software. *P* values of less than 0.05 were considered to be significant.

## SUPPLEMENTARY FIGURES AND TABLE



## Abbreviations

ES: Ewing sarcoma
EMT: epithelial to mesenchymal transition
miRNAs: microRNAs
3′UTR: 3′-untranslated region
hMSCs: human mesenchymal stem cells
RNA-seq: RNA-sequencing
FDR: false discovery rate
cyclin D2: CCND2
5-Aza-CdR: 5-aza-2’-deoxycytidine
TSA: histone-deacetylase inhibitor trichostain A
SPF: pathogen-free
HE: hematoxylin and eosin
