# Supplementary material for: miR-124 represses the mesenchymal features and suppresses metastasis in Ewing sarcoma
Source: Oncotarget. 2016 Dec 31;8(6):10274–86. doi: 10.18632/oncotarget.14394 (PMC5354658; doi:10.18632/oncotarget.14394)
Supplement: Supplementary file 1 [file oncotarget-08-10274-s001.pdf]

miR-124 represses the mesenchymal features and suppresses metastasis in Ewing sarcoma

SUPPLEMENTARY DATA

MATERIALS AND METHODS

Cell culture and transfection

Human ES cell lines (A673, SK-ES-1, RD-ES, SK-N-MC) were obtained from the American Type Culture Collection. These cell lines were immediately expanded and frozen such that they could be restarted every 3 to 4 months from a frozen vial of the same batch of cells. Human mesenchymal stem cells (MSCs) were isolated from normal adult human bone marrow withdrawn from bilateral punctures of the posterior iliac crests of three normal volunteers. MSCs were maintained at low confluency in IMDM, 10% FBS, and PDGF-BB (10 ng/ml). All cell lines were authenticated (Hybribio Bioscience & Technology Inc., Guangzhou, China). A673 and RD-ES cells were maintained in RPMI 1640 medium (PAA, Austria) supplemented with 10% fetal bovine serum (FBS; PAA, Austria), streptomycin (100 μg/ml), and penicillin (100 U/ml). SK-ES-1 cells were propagated in McCoy's 5A medium (Invitrogen, Carlsbad, CA, USA), supplemented with 10% FBS. SK-N-MC cells were maintained in DMEM medium, supplemented with 10% FBS. All cells were incubated in a humidified atmosphere of 5% CO_2 at 37°C as previously reported (30). MiR-124/scramble mimics were purchased from Dharmacon (Austin, TX, USA). According to manufacturer's instructions, all oligonucleotides were transfected into ES cells to a final concentration of 50 nM by Dhamafect 1 (Dharmacon, Lafayette, CO, USA). Cells were collected for further experiments 48 h post-transfection.

Differentiation assays

For adipogenic differentiation, cells were plated and grown for two days at 37°C, 5% CO2 in RPMI 1640 or McCoy's 5A medium with 10% FCS, 0.5 mM isobutylMethylXanthine (Sigma, American), 1 μM decamethasone (Sigma, American), and 10 μg/ml Insulin (Sigma, American). Medium was then replaced with medium and cells were grown for 6 days with medium changes every two days, then cells were collected. For osteogenic differentiation, cells were grown in RMPI 1640, 10% serum, 0.1 μM decamethasone (Sigma, American), 0.15 mM L-ascorbic acid (Sigma, American), 2 mM β-glycerophosphate and 0.1 mM NaH2PO4 for 21 days. For chondrogenic differentiation, the relative methods were performed as previously described [9].

Cell proliferation and cell cycle analyses

For cell proliferation analysis, cells were seeded into 24-well plates at 8×10^3 cells per well, and then incubated in 10% Cell Counting Kit-8 buffer (CCK-8, Dojindo, Japan) diluted in normal culture medium at 37°C until visible color conversion occurred. The proliferation rate was determined 0, 24, 48, and 72 h after transfection. The absorbance in each well was measured at 450 nM and 630 nM using a microplate reader. For cell cycle analysis, cells were harvested 48 h after transfection, washed twice with cold PBS, fixed in ice-cold 70% ethanol, incubated with propidium iodide and RNase A, and then analyzed by fluorescence-activated cell sorting (FACS).

All experiments were performed four times and the average percentages of cells are shown.

Cell migration and invasion assays

Migration assays were carried out in modified Boyden chambers (BD Biosciences, San Jose, CA, USA) with 8 μm pore filter inserts in 24-well plates. 24h after transfection, 2×10^5 cells suspended in serum-free medium were added to the upper chamber. Medium containing 20% FBS were added to the lower chambers as a chemoattractant. After 24 h transfection, the non-filtered cells were gently removed with a cotton swab. Filtered cells located on the lower side of the chamber were stained with crystal violet, air-dried and photographed. For analysis of invasive capacity, the transwell migration chambers were coated with Matrigel (BD Biosciences, San Jose, CA, USA) and incubated at 37°C for 3 h, allowing it to solidify. After 24 h of transfection, 4×10^5 cells suspended in serum-free medium were added to the upper chamber. The remaining steps were the same as migration assays. Three independent experiments were performed.

5-Aza-CdR and trichostatin A treatment of cell lines

ES cell lines A673 and SK-ES-1 were with 5-Aza-CdR (Sigma-Aldrich, USA) at 5 μmol/L for 3 days or 300 nmol/L trichostain A (TSA, Sigma-Aldrich, USA) for 24 hours. For the combination treatment, cells were treated with 5-Aza-CdR for 48 hours firstly. Then TSA was added, and the cells were treated for an additional 24 hours. Culture medium containing drug was replaced every 24 hours. RNA of cell lines was purified with TRIzol reagent following the instructions from manufacturer.

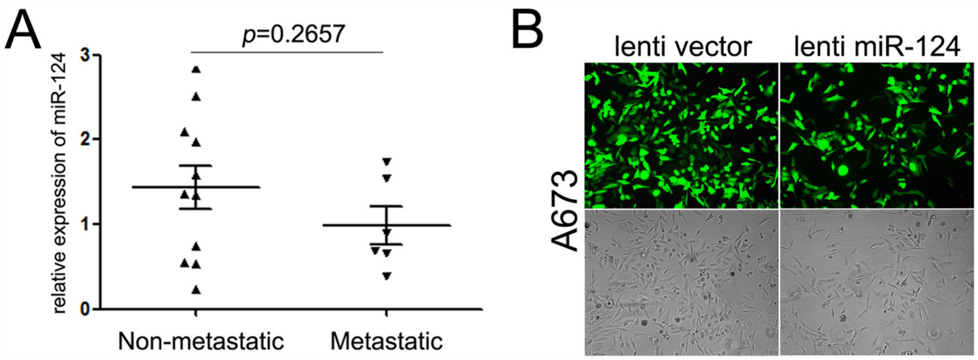

Supplementary Figure 1: Expression of miR-124 between non-metastatic and metastatic patients. A. The expression of miR-124 between 5 metastatic and 12 non-metastatic Ewing sarcoma patients was detected by quantitative RT-PCR. The expression of miR-124 showed no statistical significance between the metastatic and non-metastatic group; **B.** Immunofluorescence assays were further used to detect the transfection of lenti-viral containing miR-124 in ES cells.

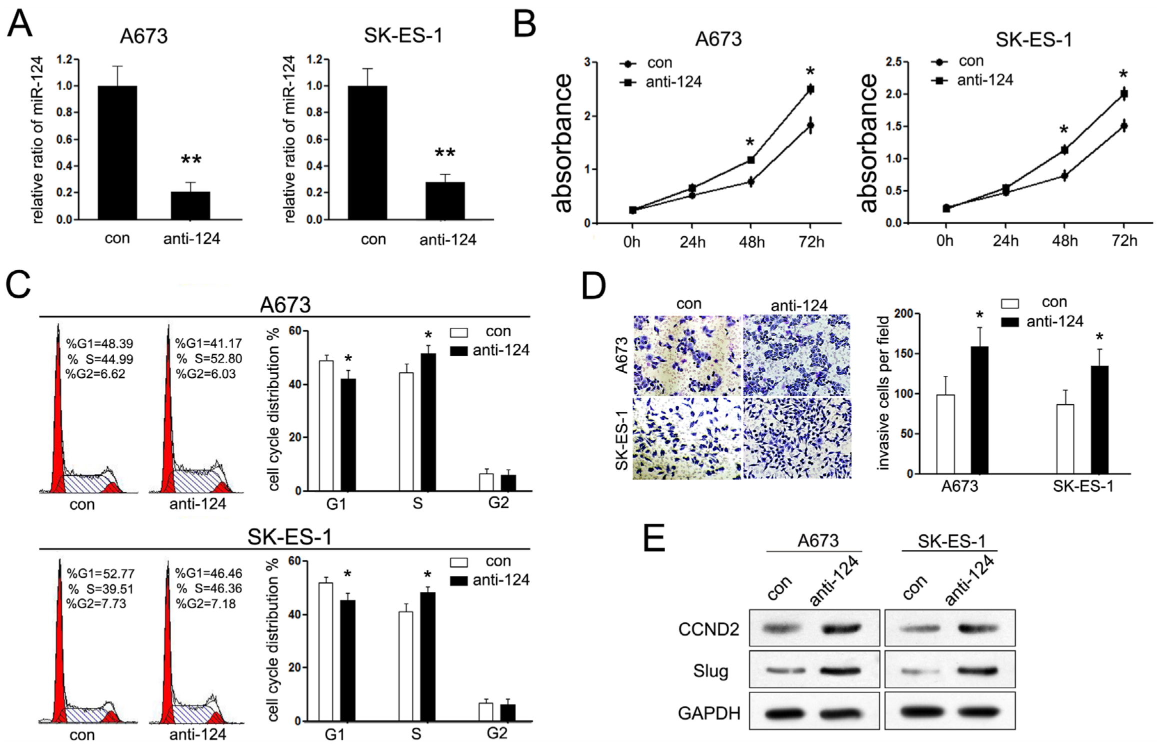

Supplementary Figure 2: Inhibition of miR-124 promoted cell proliferation, cell cycle progression and invasion of ES cells. A. RT-PCR was performed to detect the expression of miR-124 in ES cell lines (A673 and SK-ES-1) upon transfection with anti-miR-124 mimic. The expression of miR-124 was suppressed in ES cells transfected with anti-miR-124; **B.** CCK-8 assays were performed to analyze the effect of anti-miR-124 on cell proliferation of both cell lines. Suppression of miR-124 promoted cell proliferation of ES cells; **C.** Cell cycle analysis, by fluorescence-activated cell sorting (FACS) at 48h after transfection, were performed to analyze the effect of anti-miR-124 on cell cycle progression of both cell lines. Inhibition of anti-miR-124 up-regulated the cell number of S phase and down-regulated the cells of G1 phase; **D.** The effects of anti-miR-124 on cell invasion were detected using trans-well chamber assays (×400). Inhibition of miR-124 promoted cells passing through the chambers. **E.** Western blot analysis of the expression of SLUG and CCND2 upon treatment with anti-miR-124 mimic in ES cells. The data are representative of three independent experiments. Error bars represent s.e.m. *P<0.05; **P<0.01.

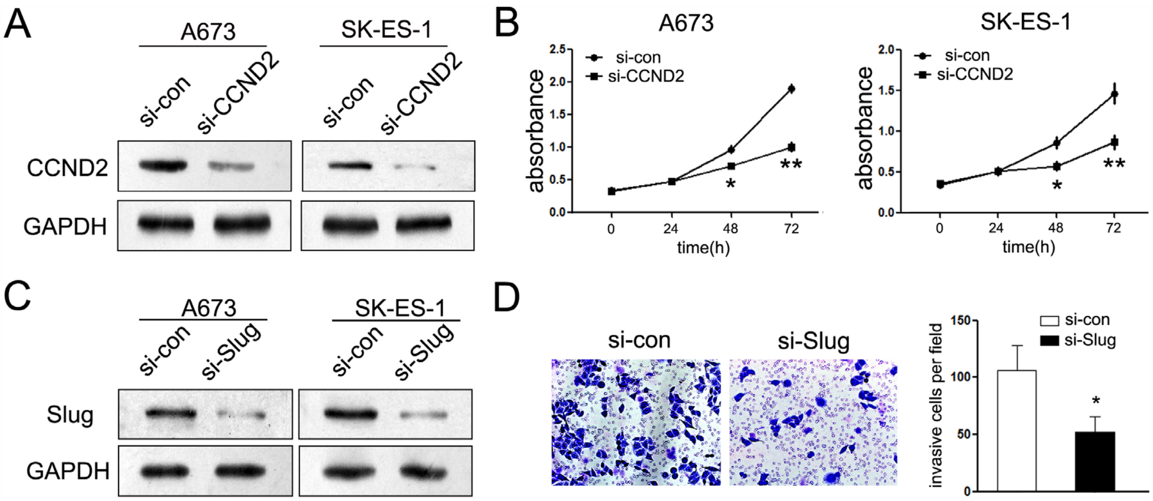

Supplementary Figure 3: SLUG and CCND2 are both target genes of miR-124. A. Western blot analysis showed the expression level of CCND2 protein in ES cells treated with siRNAs for CCND2. The expression of CCND2 was suppressed in cells transfected with siRNA; **B.** CCK-8 assays were used to detect to explore the effects of si-CCND2 on proliferation of ES cells; Suppressing the expression of CCND2 inhibited the cell proliferation of both cell lines. **C.** Western blot analysis showed the expression level of SLUG protein in ES cells treated with siRNAs for SLUG. The expression of SLUG was suppressed in cells transfected with si-SLUG; **D.** Transwell assays were performed to detect the effects of SLUG on cell invasion of ES cells that have been treated with si-SLUG (×400). Inhibiting the expression of SLUG suppressed the invasion of ES cells; The data are representative of three independent experiments. Error bars represent s.e.m. **P*<0.05; ***P*<0.01.

Supplementary Table 1: Primer sequences for PCR or Reverse transcription. F for forward, R for reverse

Gene	Primer Sequence
Primers for Reverse transcription	
miR-124	5′-GTCGTATCCAGTGCAGGGTCCGAGGTATTCGCACTGGGGCATTC-3′
U6	5′-AAAATATGGAACGCTTCACGAATTTG-3′
Slug	5′-TTTTTTTTTTTTTTTTTT-3′(Oligo(dT))
CCND2	5′-TTTTTTTTTTTTTTTTTT-3′(Oligo(dT))
GAPDH	5′-TTTTTTTTTTTTTTTTTT-3′(Oligo(dT))
Primers for quantitative Real-time PCR	
miR-124-F	5′-GCGCTAAGGCACGCGGT-3′
miR-124-R	5′-CAGTGCAGGGTCCGAGGT-3′
U6-F	5′-CTCGCTTCGGCAGCACATATACT-3′
U6-R	5′-ACGCTTCACGAATTTGCGTGTC-3′
Slug-F	5′-CACTGTGTGGACTACCGCT-3′
Slug-R	5′-TGGAGGAGGTGTCAGATGG-3′
GAPDH-F	5′-TCAACGACCACTTTGTCAAGCTCA-3′
GAPDH-R	5′-GCTGGTGGTCCAGGGGTCTTACT-3′
CCND2-F	5′-GATGCTGGAGGTCTGTGAG-3′
CCND2-R	5′-TGATGGAGTTGTCGGTGTAAA-3′
Primers for 3’-UTR	
Slug-up	5′-GAGTCTGTAATAGGATTTCCC-3′
Slug-down	5′-GGGAAATCCTATTACAGACTC-3′
CCND2-up	5′-GGAGAAAGCCAAGGGCAGT-3′
CCND2-down	5′-CTGACCAAACAGACACCCAC-3′
